# Metagenomic analyses reveal phylogenetic diversity of carboxypeptidase gene sequences in activated sludge of a wastewater treatment plant in Shanghai, China

**DOI:** 10.1007/s13213-013-0704-z

**Published:** 2013-08-28

**Authors:** Hao Jin, Bailin Li, Xu Peng, Lanming Chen

**Affiliations:** 1 Key Laboratory of Quality and Safety Risk Assessment for Aquatic Products on Storage and Preservation (Shanghai), China Ministry of Agriculture, Engineering Centre for Quality Control and Risk Assessment of Aquatic Products, College of Food Science and Technology, Shanghai Ocean University, 999 Hu Cheng Huan Road, Shanghai, 201306 People’s Republic of China; 2 Department of Biology, Copenhagen University, Ole Maaløes Vej 5, 2200 N Copenhagen, Denmark

**Keywords:** Carboxypeptidase, Phylogenetic diversity, Activated sludge, Culture-independent approach

## Abstract

Activated sludge of wastewater treatment plants carries a diverse microflora. However, up to 80–90 % of microorganisms in activated sludge cannot be cultured by current laboratory techniques, leaving an enzyme reservoir largely unexplored. In this study, we investigated carboxypeptidase diversity in activated sludge of a wastewater treatment plant in Shanghai, China, by a culture-independent metagenomic approach. Three sets of consensus degenerate hybrid oligonucleotide primers (CODEHOPs) targeting conserved domains of public carboxypeptidases have been designed to amplify carboxypeptidase gene sequences in the metagenomic DNA of activated sludge by PCR. The desired amplicons were evaluated by carboxypeptidase sequence clone libraries and phylogenetic analyses. We uncovered a significant diversity of carboxypeptidases present in the activated sludge. Deduced carboxypeptidase amino acid sequences (127–208 amino acids) were classified into three distinct clusters, α, β, and γ. Sequences belonging to clusters α and β shared 58–97 % identity to known carboxypeptidase sequences from diverse species, whereas sequences in the cluster γ were remarkably less related to public carboxypeptidase homologous in the GenBank database, strongly suggesting that novel carboxypeptidase families or microbial niches exist in the activated sludge. We also observed numerous carboxypeptidase sequences that were much closer to those from representative strains present in industrial and sewage treatment and bioremediation. Thermostable and halotolerant carboxypeptidase sequences were also detected in clusters α and β. Coexistence of various carboxypeptidases is evidence of a diverse microflora in the activated sludge, a feature suggesting a valuable gene resource to be further explored for biotechnology application.

## Introduction

Activated sludge is used for treating sewage and industrial wastewaters, and it is the most common biological wastewater treatment technology used in industrialized countries (Kim et al. 2010). Sewage and industrial wastewater contain high amounts of complex organic matter which is mainly in the form of proteins and lipids (Raunkjær et al. 1994), and often also nitrogen (as ammonium and organic nitrogen) and phosphorus. The biomass that reduces the complex content of the sewage is the activated sludge, biological flocs mainly composed of saprotrophic bacteria, and some protozoa and small metazoans (Wilén et al. 2008). Microorganisms not only take up some small molecules for intracellular metabolism but also enzymatically hydrolyze a large fraction of the organic matter through a series of hydrolytic reactions to smaller units, which can be taken up by the bacterial cell uptake system (Gessesse et al. 2003). Previous research indicated that activated sludge is a good source for studying and discovering proteases (Gessesse et al. 2003), which play a vital role in the extracellular catabolism of organic matter in activated sludge. As one important group of proteases, carboxypeptidases (EC number 3.4.16–3.4.18) hydrolyze a peptide bond of amino acid residues at the carboxyl-terminal (C-terminal) end of a protein or peptide. They have diverse functions ranging from catabolism to protein post-translational modification and regulation of biological processes (Neurath and Walsh 1976). Based on their active sites of catalytic reactions, carboxypeptidases are usually classified into metallo-carboxypeptidases (EC number 3.4.17), serine carboxypeptidases (EC number 3.4.16), and cysteine carboxypeptidase (or thiol carboxypeptidases) (EC number 3.4.18).

However, up to 80–90 % of the microorganisms detected in activated sludge by 16S rRNA genes based on molecular taxonomic studies cannot be cultured using standard cultivation techniques (Amann et al. 1995), leaving a potentially valuable resource largely unexplored. Recently, metagenomic technology has been used successfully to obtain microbial products from uncultivated microorganisms in various environments (Amann et al. 1995; Chu et al. 2008; Lee et al. 2008). Genes encoding novel enzymes, such as cellulases, chitinases, amylases, and lipases, have been screened from metagenomic libraries (Cottrell et al. 1999; Ferrer et al. 2005; Hong et al. 2007). Nevertheless, little if anything has been done to systematically investigate carboxypeptidases in microbial communities of complex environments. In this study, we focused on the diversity of carboxypeptidases in activated sludge of wastewater treatment systems in Shanghai, China, by a culture-independent metagenomic approach. Based on conserved domains including active site signature sequences of known carboxypeptidases in the GenBank database, a set of CODEHOPs was designed to amplify carboxypeptidase gene sequences in the metagenomic DNA of activated sludge by polymerase chain reaction (PCR). Our results provide the first evidence of a substantial level of carboxypeptidase sequence diversity in the microbial community of activated sludge in industrial and municipal wastewater treatment.

## Materials and methods

### Bacterial strains, plasmids and culture conditions

*Escherichia coli* TOP10 [genotype: F¯ *mcr*A **∆**(*mrr*-*hsd*RMS-*mcr*BC) Φ80 *lac*Z **∆**M15 **∆** *lac*X74 *rec*A1 *ara*D139 **∆**(*ara*-*leu*)7697 *gal*U *gal*K *rps*L (Str^R^) *end*A1-*nup*G] (TianGen Biotech, Beijing, China) was used as a host strain for transformation of recombinant plasmids in the cloning. Plasmid pGM-T (TianGen) was employed as a cloning vector for PCR products. *E. coli* was routinely grown in Luria-Bertani (LB) medium (Sambrook and Russell 2001) aerobically at 37 °C, while recombinant *E. coli* strains were grown in LB medium in both liquid broth and agar plates supplemented with amplicillin (100 μg/ml).

### Extraction of metagenomic DNA from activated sludge

Samples of activated sludge were collected from a wastewater treatment plant located in Lingang, Pudong District, Shanghai, China, where industrial and municipal wastewater was treated. Extraction of metagenomic DNA was carried out immediately after samples had been transported on ice to the laboratory in Shanghai Ocean University (Shanghai, China). Metagenomic DNA was isolated according to the method of in situ lysis of microorganisms described by Zhou et al. (1996) with some modifications. Briefly, a sample (50 g) of wet activated sludge and 135 ml DNA extraction buffer [100 mmol/l Tris-HC1, 100 mmol/l EDTA, 1.5 mol/l NaC1 and 1 % (w/v) hexadecyltrimethylammonium bromide, pH 8.0; Sigma-Aldrich, MO, USA)] were blended vigorously at 37 °C for 0.5 h. Then, 0.5 ml protease K (20 mg/ml) and 2.0 ml SDS (20 %) were added, and the mixture was incubated at 50 °C for 3 h with gentle end-over-end inversions every 20 min. The supernatant was collected after centrifugation at 6,000 *g* for 10 min at 25 °C in a high speed centrifuge (Hitachi Himac CR 21G; Hitachi Koki, Japan), and then mixed with an equal volume of chloroform:isopropyl alcohol (24:1). The aqueous phase was recovered by centrifugation at 12,000 *g* at 4 °C for 10 min, in which DNA was precipitated by adding 0.1 volume of 3 M sodium acetate (pH 5.3) and 2 volumes of chilled anhydrous ethanol, held at −20 °C for 2 h, and then recovered by centrifugation at 13,000 *g* for 15 min at 4 °C. The DNA pellet was washed with 70 % ethanol, and resuspended with 200 μl sterile Mili-Q water (Millipore, Billerica, MA, USA). DNA concentration was determined in a SAM 1000 Spectrophotometer (Merinton Technology, Beijing, China). The isolated DNA was further purified by using the Wizard DNA Clean-Up System (Promega, WI, USA), according to the manufacturer’s instruction.

### Designing of degenerate primers for PCR

Degenerate primers were designed based on public carboxypeptidase amino acid sequences in the National Center for Biotechnology Information (NCBI) database (http://www.ncbi.nlm.nih.gov/Entrez). By using the program Clustal W2 (Thompson et al. 1994) (http://www.ebi.ac.uk/Tools/msa/ClustalW2/), multiple sequence alignments were carried out with each of the three sets of carboxypeptidase sequences which were 490–511, 491–506, and 391–404 amino acids, respectively, representing different taxonomic lineages. The resulting conserved blocks of amino acid residues were used as input to load into the program iCODEHOP (Boyce et al. 2009) (https://icodehop.cphi.washington.edu/i-codehop-context/Welcome), by which degenerate primers were designed. A CODEHOP is a hybrid primer consisting of a 3′-degenerate ‘core’ and 5′-non degenerate ‘clamp’ region (Boyce et al. 2009). The default values of iCODEHOP were taken. The maximum degeneracy of 3′-‘core’ region of each CODEHOP was set at 128, while the annealing temperature of 5′-non degenerate ‘clamp’ of CODEHOP was set at 60 °C. Three sets of degenerate primers were chosen for PCR reactions in this study, the sequences of which were as following: CG-F: 5’- GTCCGTGCACCCCttytgyggngg-3′ CG-R: 5′-CCAGGGTGTAGCAGGgraartanccra-3′, CFC-F: 5′-GGTCCTGAAGCAGATCggntaygaytt-3′, CFC-R: 5′-GCCCAGGGCGTAGswnggraarta-3′, CFA-F: 5′-CCGGGCCGACATCgaygcnytncc-3′, and CFA-R: 5′-GGTGGGGCATGGAGscrtgnccncc-3′. Oligonucleotide primers were synthesized by the Shanghai Sangon Biological Engineering Technology Services (Shanghai, China).

### PCR conditions

The carboxypeptidase sequences were amplified by PCR using the purified metagenomic DNA of the activated sludge as templates. PCR amplification was performed in a 20-μl reaction volume containing 1× Premix Ex Taq Version 2.0 (Japan TaKaRa BIO, Dalian, China), 2.5 μM each of the oligonucleotide primers, and approximately 10–20 ng of template DNA. All amplifications were performed in a Peter Thermal Cycler (BIO-RAD, CA, USA).

Touchdown PCR was performed with the degenerate primer pair CG-F and CG-R under the following conditions: initial denaturation of 94 °C for 5 min was followed by 15 cycles consisting of denaturation at 94 °C for 30 s, primer annealing at 65 °C for 30 s with a gradual decrease of 1 °C per cycle, and elongation at 72 °C for 1 min, and then followed by 20 cycles consisting of 94 °C for 30 s, 55 °C for 30 s, and 72 °C for 1 min. The final extension step was at 72 °C for 10 min. The best annealing temperature of the degenerate primer pair CFC-F and CFC-R was determined by temperature gradient PCR, and established where a single band was observed on a 1.2 % agarose gel under the following conditions: initial denaturation of 95 °C for 5 min was followed by 30 cycles consisting of denaturation at 94 °C for 30 s, primer annealing at 47.2 °C for 30 s, and elongation at 72 °C for 1 min, followed by final elongation at 72 °C for 10 min. Similarly, the temperature gradient PCR was also established with the degenerate primer pair CFA-F and CFA-R under the similar conditions as described above, except the primer annealing at 58.3 °C for 30 s, and elongation at 72 °C for 45 s. The quality of the extracted DNA was tested by PCR with bacterial 16S rDNA universal primer pair 27 F (5′-AGAGTTTGATCCTGGCTCAG-3′) and 1492R (5′-TACCTTGTTACGACTT-3′). PCR reactions were carried out as following: initial denaturation of 95 °C for 5 min was followed by 30 cycles consisting of denaturation at 94 °C for 30 s, primer annealing at 55 °C for 30 s, and elongation at 72 °C for 2 min, and a final extension of 72 °C for 10 min.

A sample (5 μl) of each amplification reaction was analyzed by agarose gel electrophoresis running in a Sub-Cell GT cell (BIO-RAD) with a 1.2 % or 0.7 % agarose gel and 1× Tris-acetate-EDTA (TAE) buffer (40 mM Tris-acetic acid and 2 mM ethylenediaminetetraacetate disodium, EDTA-Na_2_). After the run, agarose gels were stained with ethidium bromide (0.33 mg/l) for 20 min. Amplified DNA fragments were visualized under short-wavelength UV light (260 nm) and imaged by the UVP EC3 Imaging System (UVP, CA, USA).

### Construction of carboxypeptidase sequence clone libraries

After PCR amplification, the desired products with 5′-A overhangs yielded by the Taq DNA polymerase were purified by agarose gel electrophoresis, and recovered from the gel by Axygen Gel Extraction Kit (Axygen, CA, USA). The purified PCR products were individually measured for DNA concentration, and ligated into the pGM-T vector with 3′ T overhangs at the insert site. The ligation reaction was preformed in a 10-μl reaction volume with a molar ratio of vector: insert of 1:3, and incubated at 16 °C overnight, according to the manufacturer’s instruction. Competent *E. coli* TOP 10 cells were transformed with the ligation DNA by the heat-shock method (Sambrook and Russell 2001). Briefly, 2 μl of each ligation DNA was added into 100 μl *E. coli* TOP 10 competent cells in a sterile 1.5-ml microcentrifuge tube, and incubated on ice for 30 min. The cells were then heat-shocked in water bath at 42 °C for 45 s, and immediately returned the tube to ice for 2 min. Subsequently, 900 μl prewarmed LB medium was added to suspend the cells, followed by incubation at 37 °C for 1 h with shaking. A sample of 100 μl of each transformation culture was spread onto LB-ampicillin plates containing 40 μl 5-bromo-4-chloro-3-indolyl-β-D-galactopyran-oside (X-gal, 20 mg/ml) and 16 μl isopropyl-β-D-thiogalactopyranoside (IPTG, 50 mg/ml). Plates were incubated overnight at 37 °C, and white colonies were randomly picked for further analysis. Transformation control was prepared with 0.1 ng of uncut plasmid pUC19.

### Sequencing and phylogenetic analyses

The white recombinant colonies were randomly picked for plasmid DNA preparation by using the MiniBEST plasmid purification kit (TaKaRa, China). Automated DNA sequencing of the insertions on purified recombinant vectors was carried out using the ABI 3730XL capillary sequencer (Applied Biosystems, CA, USA) and BigDye® terminator version 3.1 cycle sequencing kit (Perkin-Elmer, MA, USA) at the China Human Genome Centre (Shanghai, China). The universal sequencing primers, forward (5′-TGTAAAACGACGGCCAGT-3′) and reverse (5′-CAGGAAACAGCTATGACC-3′), on the pGM-T vector were utilized in the sequencing reactions. The average length of high quality sequences is 650–700 bp.

Raw DNA sequence data from both DNA strands were analyzed by using the program BioEdit (v.7.0.9, http://www.mbio.ncsu.edu/BioEdit). The sequencing reads were processed to trim usual poor-quality sequence at the 3′ end of each read. The reads from both DNA strands were overlapped and assembled into full length contigs. Individual consensus sequence was exported into a FASTA format, and aligned using the Clustal W2. Putative functions were inferred by using the Basic Local Alignment Search Tool (BLAST) ( http://www.ncbi.nlm.nih.gov/BLAST). The neighbor-joining method in the molecular evolutionary genetics analysis software MEGA4 (v.4.0) (Tamura et al. 2007) was used to construct a phylogenetic tree. A bootstrap analysis with 1,000 replicates was carried out to check the robustness of the tree.

### Nucleotide sequence accession numbers

The representative carboxypeptidase sequences obtained in this study have been deposited in the GenBank sequence database under the accession numbers from JN811663 to JN811672.

## Results and discussion

### Degenerate primers targeting conserved domains of carboxypeptidases

**Fig. 1:**
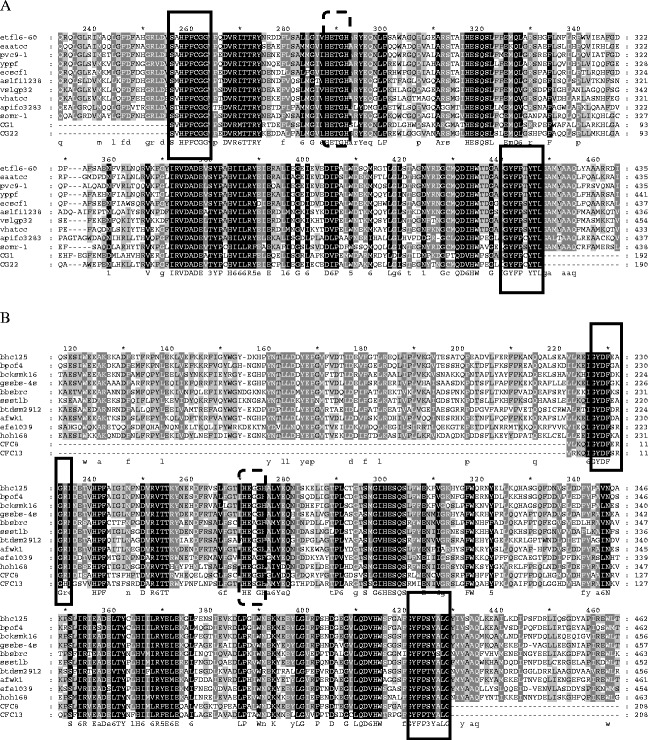

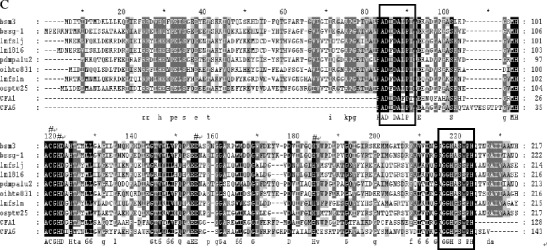
Alignments of carboxypeptidase amino acid sequences from homologues in the GenBank, and representative novel sequences obtained in this study by PCR amplification of metagenomic DNA from activated sludge samples with newly designed degenerate primers. The numbering of sequences is indicated above the alignments. The starting and ending residue numbers of each protein sequence used in this study are indicated for each sequence. Conserved amino acid residues are highlighted and shown below each alignment, and those for designing CODEHOPs are *boxed* with *solid lines*. Common active site sequence motifs are *boxed* with *dotted lines*, and conserved feature residues are located with *hash marks* above the aligned sequences. The carboxypeptidase sequences displayed in the alignments are as following: **a** *Edwardsiella tarda* FL6-60 (ADM41696.1; etfl6-60), *Erwinia amylovora* ATCC 49946 (YP_003538882.1; eaatcc), *Pantoea vagans* C9-1 (YP_003931068.1; pvc9-1), *Yersinia pestis* Pestoides F (ABP39239.1; yppf), *Enterobacter cloacae* SCF1 (ADO48513.1; ecscf1), *Aliivibrio salmonicida* LFI1238 (YP_002263204.1; aslfi1238), *Vibrio splendidus* LGP32 (YP_002416903.1; vslgp32), *Vibrio harveyi* ATCC BAA-1116 (YP_001445666.1; vhatcc), *Acetobacter pasteurianus* IFO 3283–01 (YP_003188184.1; apifo3283), *Shewanella oneidensis* MR-1 (NP_716995.1; somr-1). **b** *Bacillus halodurans* C-125 (NP_243015.1; bhc125, *Bacillus pseudofirmus* OF4 (YP_003428150.1; bpof4), *Bacillus clausii* KSM-K16 (BAD64530.1; bcksmk16), *Geobacillus* sp. SBS-4S (BAH28805.1; gsbs-4 s), *Brevibacillus brevis* NBRC 100599 (YP_002771690.1; bbsbrc), *Solibacillus silvestris* StLB046 (BAK18141.1; ssstlb), *Bacillus tusciae* DSM 2912 (YP_003589815.1; btdsm2912), *Anoxybacillus flavithermus* WK1 (ACJ33726.1; afwk1), *Enterococcus faecium* E1039 (ZP_06674096.1; efe1039), *Halothermothrix orenii* H 168 (ACL69173.1; hoh168). **c** *Bacillus sp.* m3-13 (ZP_07707682.1; bsm3), *Bacillus sp.* SG-1 (ZP_01858539.1; bssg-1), *Listeria monocytogenes* FSL J1-175 (ZP_05388672.1; lmfslj), *Listeria monocytogenes* 1816 (EGF39295.1; lm1816), *Planococcus donghaensis* MPA1U2 (ZP_08095878.1; pdmpa1u2), *Oceanobacillus iheyensis* HTE831 (NP_693505.1; oihte831), *Listeria monocytogenes* FSL N1-017 (ZP_07073423.1, lmfsln), *Ornithinibacillus sp.* TW25 (ZP_08783624.1, osptw25). The CG1 and CG22, CFC8 and CFC13, CFA1 and CFA6 were obtained from CG, CFC, and CFA libraries in this study, respectively

The carboxypeptidase sequences were searched in the NCBI database, three sets of which were used for designing the CODEHOPs. A multiple alignment of eight to ten public carboxypeptidase sequences was carried out for each set by using the Clustal W2. Two blocks of highly conserved amino acid residues HPFCG and FGYFP, GYDF and GYFP, and DALPI and GGH were identified within the multiple alignments, respectively (Fig. 1). A set of CODEHOPs was yielded after individual Clustal W2 alignment was input into the iCODEHOP. The relative quality of individual CODEHOPs was further analyzed based on the meta-data about each primer pool created by the iCODEHOP. The degenerate primer pairs CG-F and CG-R, CFC-F and CFC-R, and CFA-F and CFA-R were chosen to amplify carboxypeptidase sequences from the metagenomic DNA of the activated sludge, with an expected size of 582, 626, and 396 bp, respectively.

### Cloning of carboxypeptidase gene sequences from the metagenomic DNA extracts

The quality of the extracted metagenomic DNA was tested by PCR with the bacterial 16S rDNA universal primers 27F and 1492R. A single clear band of approximately 1.5 kb was detected by electrophoresis on a 0.7 % agarose gel, indicating good quality of the isolated metagenomic DNA from activated sludge (data not shown).

**Fig. 2:**
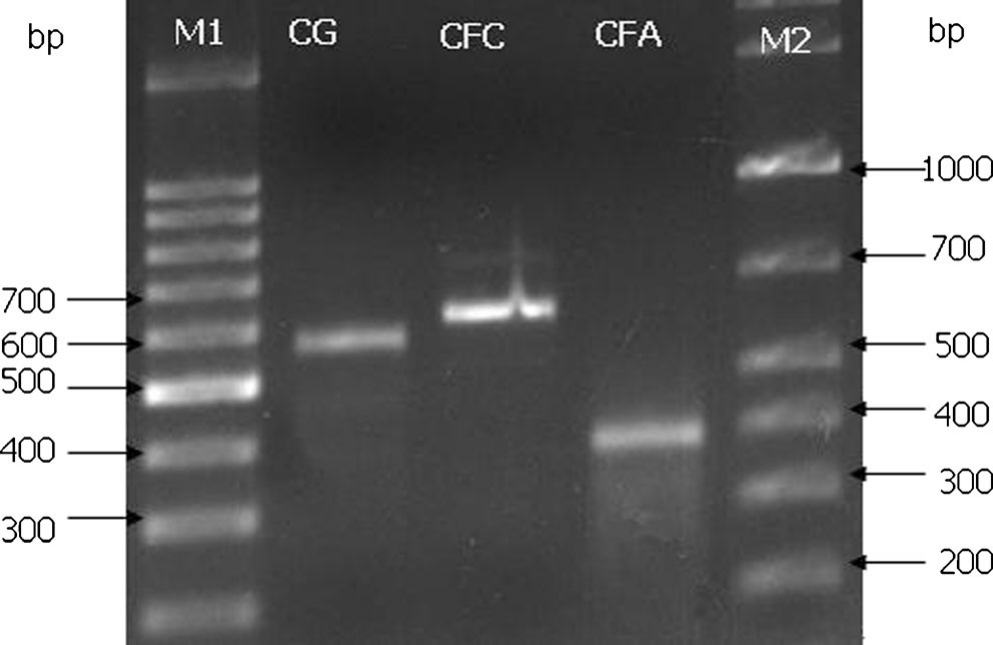
Agarose gel electrophoresis analysis of PCR products amplified by degenerate primers with metagenomic DNA isolated from activated sludge samples as templates. *M1* 1-kb plus DNA ladder, *M2* 100-bp DNA ladder. The CG, CFC, and CFA are amplicons yielded by the degenerate primers CG-F and CG-R, CFC-F and CFC-R, and CFA-F and CFA-R, respectively

The touchdown PCR reactions were performed with the degenerate primer pair CG-F and CG-R, and the desired amplicon with a length of near 0.6 kb was observed by electrophoresis on a 1.2 % agarose gel (Fig. 2). An amplicon in size between 0.6 and 0.7 kb was obtained by temperature gradient PCR with the degenerate primer paire CFC-F and CFC-R at an annealing temperature of 47.2 °C, similarly a 0.4-kb product was also obtained with the CFA-F and CFA-R at 58.3 °C (Fig. 2).

Each of three amplicons was purified, ligated into the pGM-T vector, and transformed into *E. coli* TOP10 competent cells, which yielded three carboxypeptidase sequence clone libraries, designated as CG, CFC, and CFA, respectively. About 50 white colonies from each of the three libraries were randomly picked for plasmid preparation and sequencing analyses. More than 90 % of recombinant vectors of the three libraries carried inserts in size similar to that of their corresponding amplicons by agarose gel electrophoresis analyses (data not shown), indicating the validity of the constructed libraries.

### Diversity of carboxypeptidase gene sequences in the microbial community of the activated sludge

**Fig. 3:**
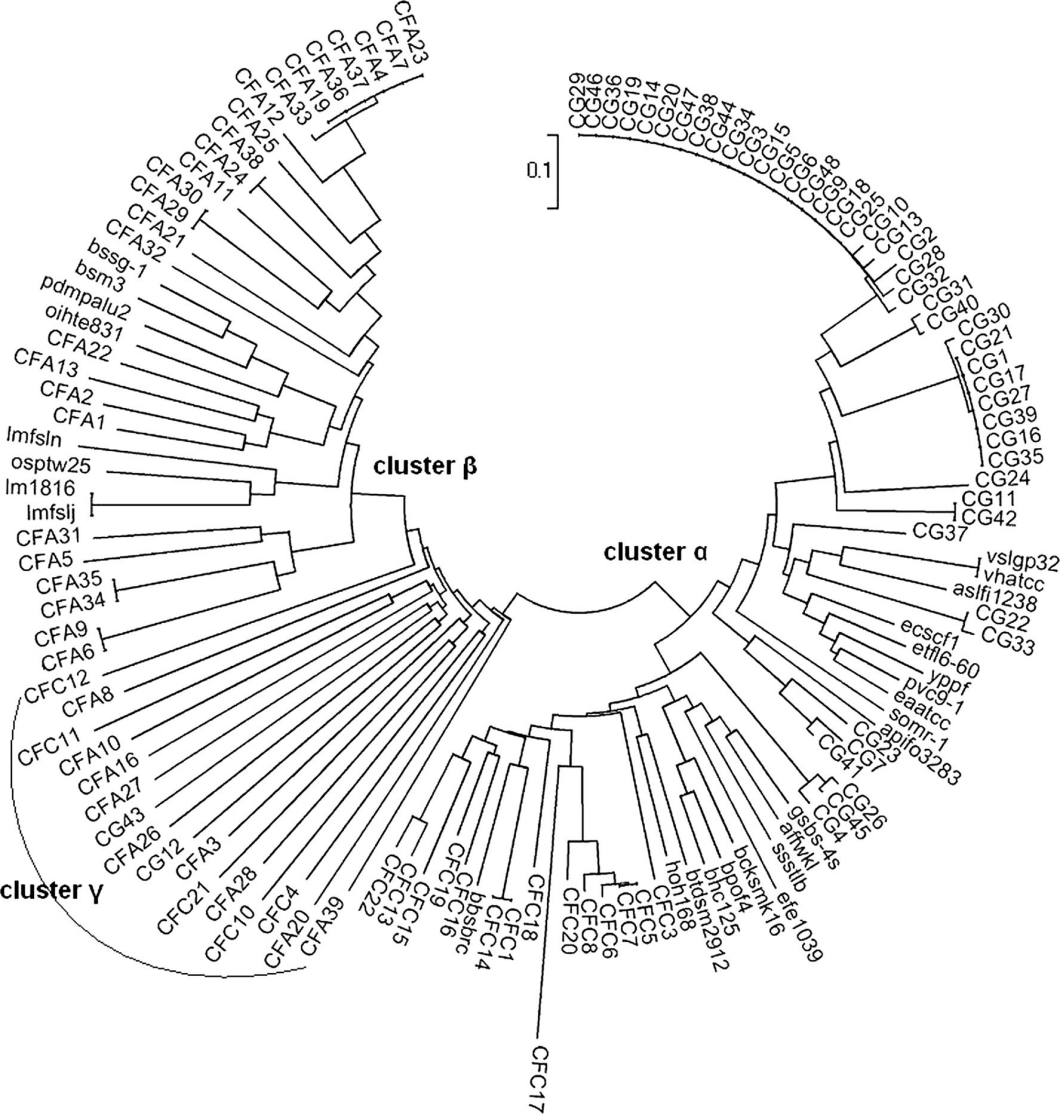
Phylogenetic tree of deduced carboxypeptidase amino acid sequences obtained from microbial community of activated sludge samples in industrial and municipal wastewater. Based on the 102 novel sequences ranged in size from 127 to 208 amino acid residues, the neighbor-joining tree was constructed by the MEGA 4.0 together with a selected set of the public carboxypeptidase sequences. The length of the branches indicates the divergence among the amino acid sequences

In total, 102 sequence contigs in high quality were obtained from the three libraries. The evolutionary relationships between these sequences and public carboxypeptidase were assessed by phylogenetic analyses. Based on the deduced amino acid sequences ranging from 127 to 208 residues, a phylogenetic tree was constructed by using the MEGA4, and it revealed three distinct clusters, designated as clusters α, β, and γ (Fig. 3). Cluster α contained sequences derived from the libraries CG and CFC, whereas cluster β consisted of sequences solely from the library CFA, and cluster γ was composed of sequences from all three libraries.

More than half of the sequences fell into cluster α, the majority of which were yielded from the library CG, while about one-fourth were from the library CFC. All the public carboxypeptidase sequences used for designing the degenerate primers CG-F/R and CFC-F/R were distributed in cluster α, but belonged to two notably different subclusters (Fig. 3). In cluster α, sequences from the library CG shared 68–97 % identity at the amino acid level with carboxypeptidase sequences from different genera including *Nitrosomonas*, *Leptothrix*, *Aeromonas*, and *Mesorhizobium*, while the sequences from the library CFC showed 57–84 % identity to the homologous from *Candidatus* Nitrospira, *Chitinophaga*, *Herpetosiphon*, *Anaerolinea*, *Rhodothermus*, and *Planctomyces*. There was a significant number of amino acid residues conserved in the multiple sequence alignments with sequences in cluster α, which spanned a common active site sequence motif His-Glu-Xaa-Xaa-His (HEXXH) (Fig. 1a, b), characteristic of the peptidase M32 family, a subclass of metallocarboxypeptidases distributing mainly in bacteria and archaea. The two histidines coordinated a divalent cation such as Zn^2+^ or Co^2+^, while a downstream glutamate polarized and oriented a zinc-coordinating water molecule during catalysis (Lee et al. 2009; Matthews et al. 1974). In addition, in cluster α, the sequences CFC1, CFC5, CFC6, CFC7, CFC8 (JN811665), CFC14, CFC17, and CFC20 displayed 59–84 % identity to the carboxypeptidase of a nitrite-oxidizing bacterium Candidatus Nitrospira defluvii, which was a key nitrifier in biological wastewater treatment of industrially contaminated sites and wastewater (Kostan et al. 2010). The CG4, CG26 (JN811671), and CG45 were closely related to the carboxypeptidase of an ammonia-oxidizing *Nitrosomonas* sp. with identities of 92 %, while the CFC13 (JN811666) and CFC22 were closely related to that from a nature’s scavenger, *Herpetosiphon aurantiacus* (Quinn and Skerman 1980), with similarity of 60 and 62 %, respectively. The CG22 (JN811664) and CG33 shared 68 and 73 % identity to the carboxypeptidase of an aerobic sheath-forming bacterium *Leptothrix cholodnii* that was often found in oligotrophic and metal-rich aquatic environments (Takeda et al. 2005). These results underline the considerable phylogenetic diversity of the carboxypeptidase gene sequences in the microbial community of the activated sludge.

Cluster β was grouped by one-fourth of the sequences, all of which were derived from the library CFA, and shared 58–78 % identity to proteases from different species, except for two sequences with less than 50 % coverage. In cluster β, most sequences were related to the peptidase M20D family, amidohydrolase, and one to the M21D family of the genera including *Haliscomenobacter*, *Runella*, *Mucilaginibacter*, *Chitinophaga, Comamonas*, *Cyclobacterium*, *Acidovorax*, *Bordetella*, *Chthoniobacter*, *Ruegeria*, and *Dyadobacter*. In addition, the CFA9 and CFA5 were close to the carboxypeptidase Ss0 subfamily protein of *Myxococcus* (YP_004669645.1), the CFA21 to the bifunctional carboxypeptidase of *Thermococcus* (YP_002306730.1), and the CFA32 to the thermostable carboxypeptidase1 of *Thermomicrobium* (YP_002523939.1). Similar to cluster α, the public carboxypeptidase sequences used for designing the CFA-F/R primers were spread in the cluster β, and numerous conserved residues were also observed in the multiple sequence alignment containing the metal binding sites of the M20 family conserved domains (Fig. 1c). The peptidase M20 family including carboxypeptidases belongs to zinc peptidases which play vital roles in metabolic and signaling pathways throughout all kingdoms of life (Rawlings and Barrett 1995). Moreover, in cluster β, the sequences CFA33, CFA34, CFA13, and CFA1 (JN811667) showed 63–75 % identities to the peptidases of *Haliscomenobacter*, *Comamonas*, and *Acidovorax* species that have been commonly observed in activated sludge samples originating from both municipal and industrial wastewater treatment plants (Kragelund et al. 2008; Kraigher et al. 2008; Srinandan et al. 2011). These results strongly reinforce the high diversity of carboxypeptidases present in the activated sludge.

A total of 16 % of the sequences fell into cluster γ, about half of which were generated from the library CFA, while the others were from the libraries CFC and CG. In contrast to the high identity and coverage (72–99 %) in clusters α and β, the deduced amino acid sequences in cluster γ remarkably displayed low levels of similarity and coverage to public homologues and hypothetical proteins in GenBank, except for CFA20, CFA8, and CFC4. CFA20 carried 48 % identity to the ATP-dependent protease La (Lon) domain protein of 218 amino acids from *Stigmatella aurantiace* DW4/3-1 (ZP_01466891.1), and CFA8 (JN811669) had 64 % identity to the glycoprotease family metalloendopeptidase of *Thermobaculum terrenum* (YP_003321788.1), while CFC4 (JN811670) showed 88 % identity to the hypothetical protein NIDE4078 of *Candidatus* Nitrospira defluvii (Lücker et al. 2010). Interesting, in theluster γ, some sequences derived from the three libraries notably had 7–30 more amino acids than the desired products amplified with three CODEHOPs, while the other sequences were slightly shorter than predicted lengths. No public carboxypeptidases sequences were grouped into cluster γ, but a common ancestor was shared among the three clusters (Fig. 3). Moreover, sequences in cluster γ seemed less close to each other than to database homologues, strongly suggesting that novel carboxypeptidase families or microbial niches existed in the activated sludge.

### Specificity of the CODEHOPs-based PCR for screening novel carboxypeptidases from complex environmental samples

The diversity and presence of different conserved motifs among carboxypeptidase families led to the fact that no primers are omnipotent for broad range-based amplification of carboxypeptidase gene sequences. This problem may be solved by designing more sets of primers specific for carboxypeptidase families. Nevertheless, the metagenomic technique is a powerful tool for evaluating phylogenetic diversity of carboxypeptidases present in activated sludge by a culture-independent approach. In this study, a large number of carboxypeptidase sequences were cloned by PCR amplification of metagenomic DNA of the activated sludge samples. The inferred amino acid sequences in three libraries had various identities to public carboxypeptidase sequences of multifarious bacterial resources, including representative strains present in the process of industrial and sewage treatment and bioremediation. In addition, the CG1 (JN811663) and CFC19 in cluster α shared 72 and 60 % identity to the thermostable carboxypeptidases of *Mesorhizobium loti* (NP_104928.1) and *Rhodothermus marinus* (YP_003290669.1), respectively, while the CFA6 (JN811668) and CFA9 in cluster β was related to that of the halotolerant *Myxococcus fulvus* (YP_004669645.1). Interesting, the CFA2 in theluster β and the CG37 (JN811672) in cluster α were closely related to the carboxypeptidases of the pathogenic bacteria belonging to *Bordetella* and *Aeromonas* species with identities of 72 and 97 %, respectively; the latter was also revealed in the activated sludge samples by 454 pyrosequencing (Ye and Zhang 2011). Moreover, some sequences in clusters α and β had 63–76 % identity to the carboxypeptidases of *Mucilaginibacter* and *Chitinophaga* species that are able to degrade pectin, xylan, chitin, laminarin, and some other polysaccharides. Taken together, our data revealed a significant diversity of carboxypeptidase sequences existing in the metagenomic DNA of the activated sludge samples, suggesting a diverse microflora in this special environment.

To our knowledge, this study constitutes the first investigation of microbial carboxypeptidase diversity in complex environments by using a culture-independent strategy. The multifunctional property of proteases is based on their diverse structures, which is a consequence of differences in protein sequences. Thus, screening for novel carboxypeptidases with various characteristics is of interest from both industrial and academic standpoints. The results in this study demonstrated the specificity and effectiveness of the sequence-based screening approach for identifying novel carboxypeptidases directly from complex environmental samples, but also expanded the views on the biodiversity and utilization of activated sludge in biotechnology application.
